# Novel Color Change Film as a Time–Temperature Indicator Using Polydiacetylene/Silver Nanoparticles Embedded in Carboxymethyl Cellulose

**DOI:** 10.3390/polym12102306

**Published:** 2020-10-08

**Authors:** Aphisit Saenjaiban, Teeranuch Singtisan, Panuwat Suppakul, Kittisak Jantanasakulwong, Winita Punyodom, Pornchai Rachtanapun

**Affiliations:** 1 Doctor of Philosophy Program in Nanoscience and Nanotechnology (International Program/Interdisciplinary), Faculty of Science, Chiang Mai University, Chiang Mai 50200, Thailand; toomtamapisit@gmail.com; 2 School of Agro-Industry, Faculty of Agro-Industry, Chiang Mai University, Chiang Mai 50100, Thailand; teeranuch.kung@gmail.com (T.S.); jantanasakulwong.k@gmail.com (K.J.); 3 Department of Packaging and Materials Technology, Faculty of Agro-Industry, Kasetsart University, 50 Phaholoyothin Rd., Chatuchak, Bangkok 10900, Thailand; panuwat.s@ku.ac.th; 4 The Cluster of Agro Bio-Circular-Green Industry (Agro BCG), Chiang Mai University, Chiang Mai 50100, Thailand; 5 Center of Excellence in Materials Science and Technology, Chiang Mai University, Chiang Mai 50200, Thailand; winitacmu@gmail.com; 6 Department of Chemistry, Faculty of Science, Chiang Mai University, Chiang Mai 50200, Thailand

**Keywords:** activation energy, biopolymer, carboxymethyl cellulose, color, intelligent packaging, nanocomposite, nanoparticles, plasticizer, polydiacetylene, time–temperature indicator

## Abstract

Time–temperature indicators (TTIs) can be important tools in product applications to monitor food quality losses, especially for fruits and vegetables. In this context, the effects of silver nanoparticles (AgNPs) and glycerol on the color change of polydiacetylene/AgNPs (PDA/AgNPs) embedded in carboxymethyl cellulose (CMC) film as time–temperature indicators (TTIs) were investigated. A CMC film prepared with 30 mg/L AgNPs and a 1:3 (v/v) PDA:AgNP ratio exhibited a faster color change than under other conditions. At 35 °C, the films with PDA/AgNPs changed color from purplish-blue to purple and purple to reddish-purple over time due to the higher thermal conductivity of AgNPs and larger PDA surface area exposed to specific temperatures. The total color difference (TCD) of PDA/AgNP-embedded CMC film directly changed with regard to time and temperature. However, adding glycerol to the system resulted in a symmetrical chemical structure, a factor that delayed the color change. Scanning electron micrographs showed AgNPs embedded in the CMC films. Transmission electron micrographs indicated a core-shell structure of PDA/AgNP vesicles in the CMC matrix. PDA/AgNP vesicles were confirmed by second derivative Fourier transform infrared spectroscopy, with a new peak at 1390–1150 cm^−1^. The kinetics of TTIs from PDA/AgNP-embedded CMC films yielded an activation energy of 58.70 kJ/mol.

## 1. Introduction

Food waste has been and continues to be a major problem; it exerts a myriad of effects on whether the use of natural resources is worthwhile and causes environmental pollution. Besides, it directly affects human life because the purchase of low-quality products causes the formation of toxic substances in food, a factor that affects the suitability of food and the safety of consumers’ lives. Therefore, the food industry is an essential part of potential solutions because it controls the food system from the beginning until delivery to consumers. Hence, the food industry requires quality control tools, including intelligent packaging, which has become a more viable packaging strategy than conventional methods. Intelligent packaging can be designed to record information during transportation, storage, communication, consumer information, and distribution. This modality is essential to continuously show the quality and safety of food based on a label that can monitor the quality of the product. Therefore, there has been and continues to be extensive research into the development of intelligent packaging to advance food quality.

Time and temperature control is an essential factor that affects food quality. Time–temperature indicators (TTIs) have received attention for their potential in control systems that ensure quality throughout a product’s life cycle [1]. TTIs are critical to advance efficient food packaging systems while assuring food safety. TTIs can help monitor food quality during transportation, storage, and distribution, including the time between disposals. TTIs allow continuous monitoring of products to ensure the safety of the consumer and overall product quality [2], a factor that is essential for consumer acceptance.

TTIs are simple, effective, and easy to use devices for monitoring, recording, and indicating the overall effect of temperature on quality from manufacturing to the end consumer [3]. Consumers can check the quality and decide to choose food products through a color response that matches the quality of food, indicative of transport conditions and storage temperature [4]. Consumers can observe the color changes of a chemical reaction from the indicator, which can be prepared from polydiacetylene (PDA) as a sensor device. PDA is a conjugated polymer with a backbone of 1,4- addition polymerization of diacetylene monomers. PDA can be made from 10,12- pentacosadiynoic acid (PCDA). This monomer consists of a hydrophilic carboxylic group and a hydrophobic long-chain hydrocarbon. PDA also self-assembles into lipid bilayer vesicles upon ultraviolet (UV) irradiation (~254 nm) or gamma irradiation, which generates the polymeric backbone by alternating the C=C and C≡C (-ene and -yne, respectively) bonds. This phenomenon leads to a color change from colorless to blue. The electronic absorption of PDA occurs via a p-to-p* transition of electrons within the linear p-conjugated backbone. The factor is a precondition for the completed topochemical polymerization of diacetylene monomers, which will self-assemble to form a specific symmetrical structure. Subsequently, PDA vesicles may change color from blue to red with environmental stimuli such as temperature. Above 60°C, side-chain strain is relieved, a phenomenon that allows PDA to move and form stable structures and subsequently change color from blue to red [5,6].

In this investigation, PDA was modified to improve its sensitivity when exposed to a temperature lower than 60°C. Adding nanoparticles such as silica dioxide (SiO_2_) [7], zinc oxide (ZnO) [8], titanium dioxide (TiO_2_) [9], silver (Ag) [10], and gold (Au) [11] enhances the strong ionic interaction with the functional groups of polymer chains [12]. Furthermore, adding plasticizers such as PEG-400, glycerol, xylitol, and sorbitol can improve the temperature sensitivity of PDA. Recent examples of new colorimetric TTIs are plasticized PDA/SiO_2_ films, which change from purple to red within 4 days when stored below 35 °C [5]. The polymeric bilayers of PCDA show a reversible color transition when anchored on ZnO nanoparticles (ZnONPs) and an irreversible transition when PCDA forms vesicles. The color-changing properties of PDA can result in more effective chemical modification using ZnONPs. The interaction between the carboxylic groups of PDA and the ZnO surface influences the PDA color change [8]. Films with PDA and silica nanocomposite embedded in poly(vinyl alcohol) (PVOH) have been explored as a new type of colorimetric TTI. In one study, a PDA/SiO_2_/PVOH film presented a clearer blue to red color change compared with PDA/PVA films without SiO_2_ [7]. Furthermore, a study of the temperature indicator of gold nanoparticles (AuNPs) in chitosan served as an easy and temperature-sensitive indicator for frozen food products. The chitosan/AuNPs changed color from gray to dark gray, an alteration that can be used to determine the quality of frozen food [13].

Most silver nanoparticles (AgNPs) have been used as active packaging to inhibit microorganisms. For example, active nanocomposite film is prepared from gelatin solutions and different AgNP concentrations. Using an active nanocomposite film with food packaging can effectively inhibit the growth of pathogenic microorganisms [14]. However, there has been increasing research interest to use AgNPs as an additive for a TTI film. Specifically, a novel PDA could be developed using AgNPs to enhance the film’s sensitivity to temperature changes. The advantages of AgNPs are good electrical [15] and thermal [16] conductivity. AgNPs are interesting because they are non-toxic to the human body at low concentrations, are small in size, have a high surface to volume ratio [17], and form many interactions with the polymer [18]. A previous study indicated that diacetylene monomers are adsorbed on the surface of AgNPs that form a bilayer structure [19]. Hence, the addition of AgNPs to PDA might provide a suitable means to sense the environment (e.g., temperature). Moreover, AgNPs have a low specific capacity, a feature that leads to excellent heat transfer.

Several types of film would be efficient in AgNP dispersion, including poly (vinylpyrrolidone) (PVP), PVOH [20], and starch [21]. Most TTIs have used PVOH as the base film because it is transparent and water-soluble, but it is expensive [22]. In this study, we used carboxymethyl cellulose (CMC) as the base film for TTIs because it is relatively inexpensive and has similar properties to PVOH. CMC is a cellulose derivative and a polysaccharide. CMC contains a hydrophobic backbone and many hydrophilic carboxyl groups. It has received much interest due to its unique properties such as transparency, hydrophilicity, non-toxic nature, biocompatible polysaccharide, biodegradability, and good film-forming ability [17]. CMC is suitable for a wide range of applications due to its a low cost [23]. Therefore, it has been used as a stabilizer polymer [24] and to ensure dispersion stability of particles [25].

Given that there has been no research on TTIs composed of PDA/AgNPs embedded in CMC film, we examined the use of AgNPs to enhance the sensitivity of PDA at low temperatures. We aimed (i) to study preparation of PDA/AgNPs embedded in CMC for film forming; (ii) to study the effect of altering the CMC:PDA/AgNP and PDA:AgNP ratios and AgNP concentration; and (iii) to determine the effect of glycerol concentration on color change at various low temperatures.

## 2. Materials and Methods

### 2.1. Materials

PCDA (molecular weight: 374.60 g/mol) was obtained from Sigma-Aldrich (St. Louis, MO, USA). CMC was purchased from Fluka (St. Louis, MO, USA). Ethanol (C_2_H_12_O_5_) and glycerol (C_3_H_8_O_3_) were obtained from Northern Chemicals and Glasswares Ltd. Part (Chiang Mai, Thailand). An AgNP solution (10–20 nm) was obtained from Prime Nano Technology (Bangkok, Thailand).

### 2.2. Preparation of PDA/AgNPs

PCDA (0.0152 g) was dissolved in 4 mL ethanol and ultrasonicated for 10 min in a sonicator bath (S 30 H, Elma, Germany) to obtain PCDA solution. The sample solution was then filtered using a nylon syringe filter (pore size 0.45 μm) to eliminate contaminants. PCDA solution was slowly dropped into 20 mL deionized water in a sonicator bath at 60–70 °C to obtain 2 mM PCDA. Next, AgNPs were diluted in deionized water to obtain 30, 60, 90, and 120 mg/L solutions at 1:3, 2:2, and 3:1 (v/v) PCDA:AgNP ratios. Finally, the AgNP solution was added into the PCDA solution to form PCDA/AgNPs, followed by ultra-sonication at 60–70 °C for 30 min in different PDCA/AgNP ratios.

### 2.3. Film Formation

CMC (2% w/v) was dissolved in hot water (80–90 °C); it served as a substrate for film formation. The PCDA/AgNP solution was mixed with CMC solution in ratios at 1:1, 2:1, and 3:1 (v/v) CMC:PDA/AgNP ratios (Table 1). Next, the sample solution was mixed with 10, 20, or 30% glycerol (used as a plasticizer). The sample solution was cast onto an acrylic plate (15.5 × 15.3 cm) and dried at 45 °C for 12 h in a hot air oven (LDO-100E, Daihan Labtech Co., Ltd., Gyeonggi-do, Korea). The dried PCDA/AgNP was cut into a small piece (3 × 3 cm) and stored at 4 °C for 24 h or until used, then placed at 25 ± 1 °C. The film samples were activated by ultraviolet (UV) light (254 nm, 17/18 W, T8, 220VAC, [L590 × D28 mm], Phillips) for 3 min to obtain PDA/AgNP-embedded CMC films. The transparent film sample became blue with the addition of PDA/AgNPs. The study also explored the effect of different amounts of glycerol (0, 10, 20, or 30%) on the color-changing ability of PDA.

### 2.4. Morphology of PDA/AgNP-Embedded CMC Films

The film samples were immersed in liquid nitrogen and fractured to ensure that the microstructure remained clean and intact. The film fractures were sputter-coated with a thin film of gold. Next, the gold-coated film was investigated using a field emission scanning electron microscope (FE-SEM, JSM-6335F) at acceleration voltages of 0.5–30 kV.

The sample solutions were dropped on a copper grid and dried at room temperature. Then, the PDA/AgNP-embedded CMC solutions were investigated by transmission electron microscope (TEM, JEM-2010, JEOL, Japan), operating at 80 kV.

### 2.5. Chemical Structure of PDA/AgNP-Embedded CMC Films

The functional groups of PDA/AgNP-embedded CMC film were characterized by Fourier transform infrared (FT-IR) spectroscopy (Tensor 27, Bruker, Germany) directly on film samples. Next, FT-IR spectra were changed to the second derivative with OPUS Spectroscopy Software.

### 2.6. Determination of the Dynamic Parameters of Indicator Films

All films were tested at four different temperatures (5, 15, 25, and 35 °C). The color of the indicator corresponds to the total color difference (TCD or ΔE) value under different temperatures. All film samples were checked every 24 h with a color meter (Minolta CR-10, Japan). The L *, a *, and b * chroma system was used to analyze the dynamic change in the indicator’s color, which is the color difference (TCD), following Equation (1):

(1) $$\Delta E={\left[{\left(\Delta L^{*}\right)}^{2}+{\left(\Delta a^{*}\right)}^{2}+{\left(\Delta b^{*}\right)}^{2}\right]}^{1/2}$$

where ∆L* is the brightness difference between initiation and each time interval; ∆a* is the redness- greenness difference between initiation and each time interval; and ∆b* is the yellowness- blueness difference between initiation and each time interval.

According to the indicator kinetics characterized by Taoukis and Labuza [1], the total color difference value X = ΔE or TCD of the indicator maybe expressed in terms of a response function as follows:

(2) $$F\left(X\right)=\left(\frac{1}{X_{0}}-\frac{1}{X_{t}}\right)=\mathrm{kt}$$

where k is the rate constant of the reaction that is correlated with temperature and t is the storage time.

Plotting a curve between the response function of TCD F(X) and time generated a straight line, and the k of different storage temperatures may be calculated from the slope. One can take the logarithm or natural logarithm on both sides of the Arrhenius function.

(3) $$\ln\;k=\frac{E_{a}}{\mathrm{RT}}+\ln A$$

Plotting a curve between ln k and 1/T generates a straight line. The activation energy (E_a_) may be calculated from the slope, and A can be determined directly from the intercept [2].

## 3. Results and Discussion

### 3.1. Color Change Behavior in PDA/AgNP-Embedded CMC Films

**Figure 1:**
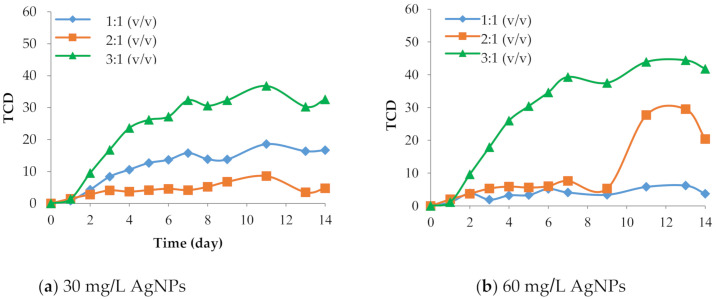

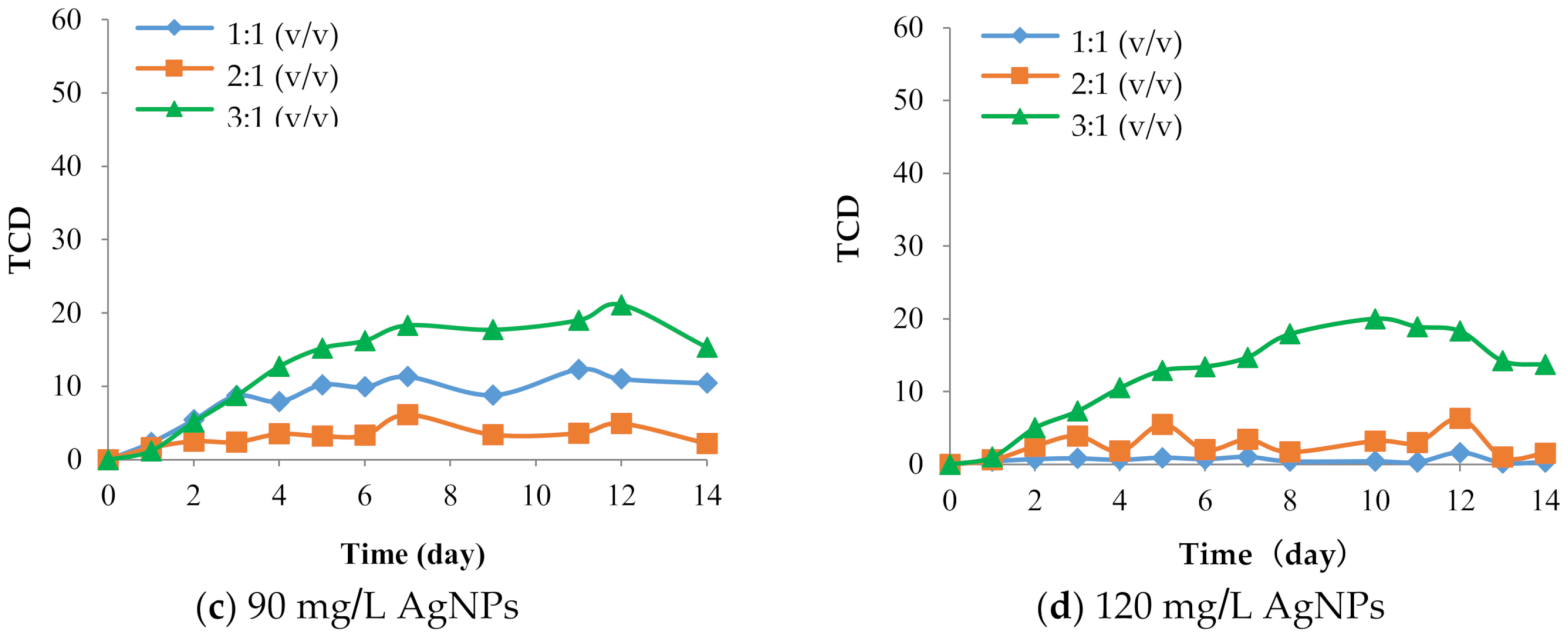
Effect of carboxymethyl cellulose:polydiacetylene/silver nanoparticle (CMC:PDA/AgNP) ratios—3:1 (green line), 2:1 (orange line), and 1:1 (blue line)—on changes in total color difference (TCD) of films at 35 °C with (**a**) 30 mg/L, (**b**) 60 mg/L, (**c**) 90 mg/L, and (**d**) 120 mg/L AgNP concentrations.

The film prepared with a 3:1 (v/v) CMC:PDA/AgNP ratio exhibited a greater color change (green line in Figure 1) compared with the other ratios because it had the smallest amount of PDA relative to CMC. The matrix of the film influences the optical and sensing properties of PDA, which are largely dependent on intramolecular noncovalent interactions [26]. Thus carboxyl groups from PDA interacted with carboxyl groups from CMC via hydrogen bonding. The reduction of CMC leads to an increase in the area of the side chain movement of PDA [27]. Moreover, the interaction is not strong because PDA can easily move and change color [19]. With regard to the AgNP concentration, 30 and 60 mg/L promoted faster color change than the higher concentrations at the 3:1 (v/v) CMC:PDA/AgNP ratio (Figure 1). Therefore, we selected 30 and 60 mg/L AgNP concentrations to further study the PDA:AgNP ratio.

**Figure 2:**
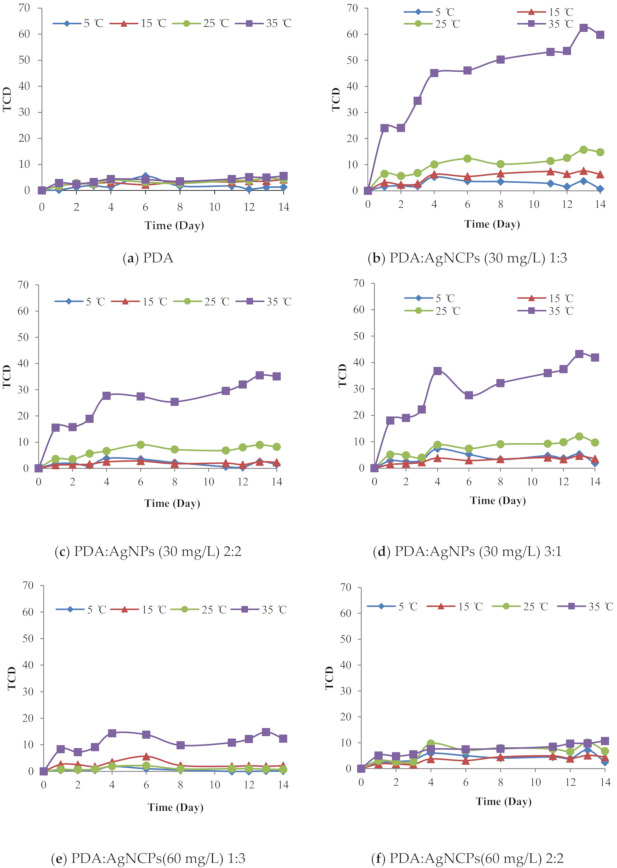

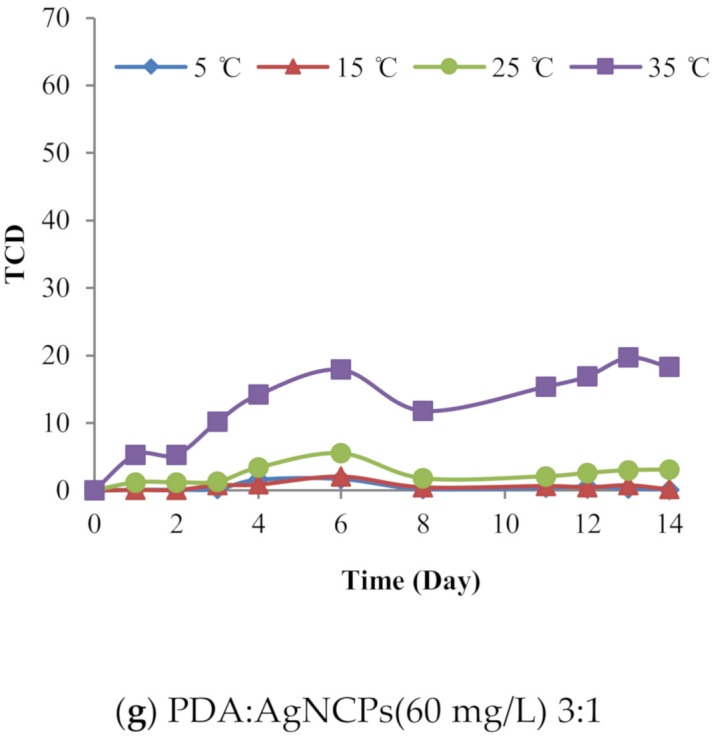
Effect of temperatures (5, 15, 25, and 35 °C) on changes in total color difference (TCD) at different polydiacetylene:silver nanoparticle (PDA:AgNP) ratios (as % of solid) and AgNP concentrations (30 and 60 mg/L): (**a**) PDA alone, (**b**) 1:3 PDA:AgNPs (30 mg/L AgNPs), (**c**) 2:2 PDA:AgNPs (30 mg/L AgNPs), (**d**) 3:1 PDA:AgNPs (30 mg/L AgNPs), (**e**) 1:3 PDA:AgNPs (60 mg/L AgNPs), (**f**) 2:2 PDA:AgNPs (60 mg/L AgNPs), and (**g**) 3:1 PDA:AgNPs (60 mg/L AgNPs) embedded in CMC:PDA/AgNPs at 75:25.

There were no significant TCD changes in the film samples at 5, 15, 25, and 35 °C as time increased. The greatest color changes occurred at 35 °C (Figure 2). The PDA/AgNP-embedded CMC film (prepared with 30 mg/L AgNPs) changed from purplish-blue to purple and purple to reddish- purple as the temperature increased. At the highest temperature, PDA mobility increased and it change color faster than at lower temperatures [5]. The PDA without AgNPs presented a slower color change than PDA with AgNPs because the PDA interacts with the surface of AgNPs [28]. AgNPs are good thermal conductors and likely more uniformly expose PDA to the specific exposure temperature [16]. Besides, the PDA/AgNP-embedded CMC film (prepared with 30 mg/L AgNPs) had a higher TCD than the PDA/AgNP-embedded CMC film (prepared with 60 mg/L AgNPs). Overall, the lower AgNP concentration had a greater influence on the film’s color change, probably because there is more water to form an AgNP and PDA core-shell structure. In the PDA/AgNP solutions, there are interactions between the –COOH group and –OH groups on the AgNP surfaces. With a lower AgNP concentration, there are more free areas to increase a packing arrangement of symmetrical chemical or core-shell structures. In addition, more core-shell structures on the thin-film sample reduce the space between the PDA chains and increase the success of polymerization. Monomer spacing is essential, primarily to facilitate the van der Waal’s forces between the pendant alkyl chains that allow the diacetylene backbone to form [29]. The improved color change efficacy demonstrated that polymerization had succeeded.

The film with the 1:3 (v/v) PDA:AgNP ratio showed the greatest color change. As the amount of AgNPs decreases, there are fewer opportunities to form the PDA/AgNP core-shell structures. A previous study examined the surface coverage density (SD) of PDCA/AgNPs, expressed in terms of the number of coating molecules per core [19]. At the 1:3 (v/v) PDA:AgNP ratio, PDA was unable to interact with itself and thus could change color. By contrast, with the other PDA:AgNP ratios, PDA was able to interact with itself and become a bulky structure. Consequently, the color change was slow, as described in the next section.

**Figure 3:**
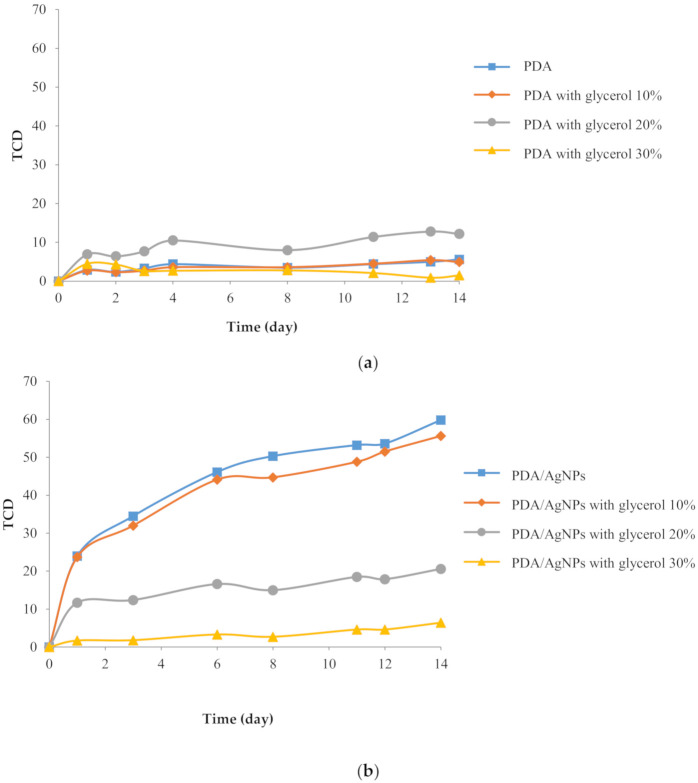
Changes in the total color difference (TCD) of (**a**) polydiacetylene (PDA) with different amounts of glycerol and (**b**) PDA/silver nanoparticles (AgNPs) with different amounts of glycerol. The compounds were embedded in CMC films at a 1:3 PDA:AgNP ratio (prepared with 30 mg/L AgNPs) and at 35 °C.

Over 14 days, the PDA-embedded CMC film did not change color (Figure 3a). However, the PDA/AgNP-embedded CMC film exhibited increased color change (Figure 3b) because adding AgNPs separated bulky PDA structures; PDA was adsorbed on the surface of AgNPs. Moreover, AgNPs are good thermal conductors, and thus the PDA was more uniformly exposed to the specific exposure temperature. This factor promoted a definite color change.

**Figure 4:**
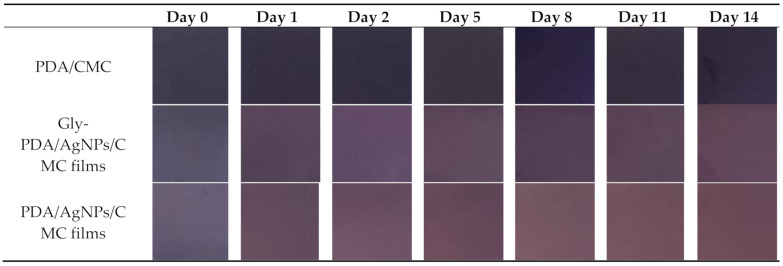
Color change comparisons of polydiacetylene (PDA)-embedded, PDA/silver nanoparticle (AgNP)-embedded, and glycerol (Gly)-PDA/AgNP-embedded carboxymethyl cellulose (CMC) films.

In contrast, when the glycerol concentration increased, the PDA color change decreased. The glycerol, a small molecule, could be inserted between polymer chains to interrupt the formation of polymer (–COOH of PDA)-polymer (CMC) hydrogen bonds [30]. So, twisting of the PDA side chains was blocked by glycerol. Moreover, the glycerol could insert into the channel between PDA side chains. When the film samples were dried in a hot air oven, the vesicles were packed between PCDA and the glycerol [31]. When the sample film was activated with UV light, the polymerization was lower because the PCDA chain was blocked by the inserted glycerol between PDA molecules. Glycerol decreased the mobility of PDA at various temperatures (Figure 3b). Moreover, glycerol exhibits a symmetrical chemical structure that leads to decreased core-shell structure of PDA/AgNPs in CMC films [5]. Figure 4 shows the color change of each film.

**Figure 5:**
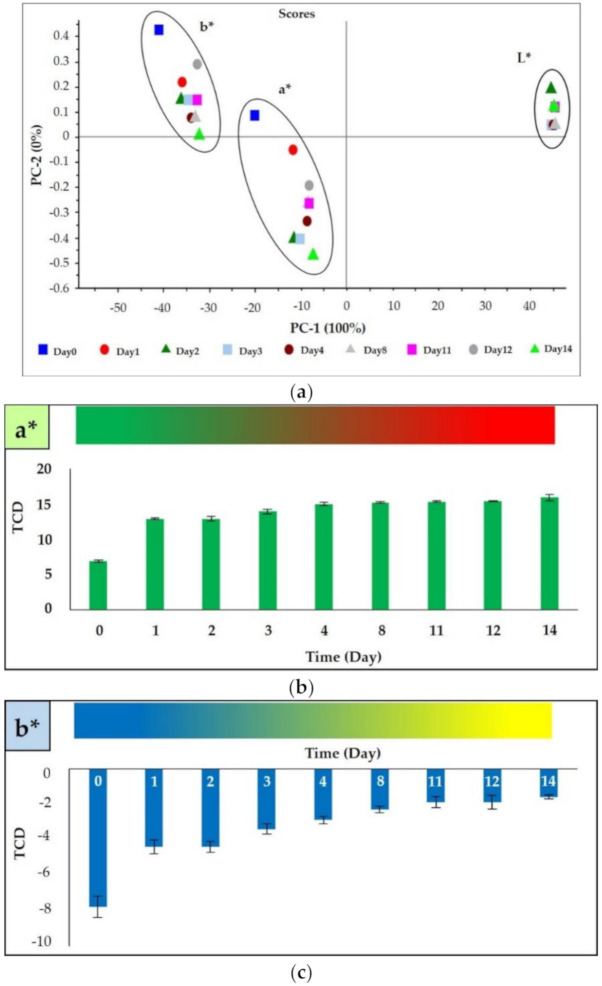
(**a**) Principal component analysis (PCA) of changes in L*, a*, and b* and bar graphs showing color change of (**b**) a* and (**c**) b* of 1:3 polydiacetylene (PDA):silver nanoparticles (AgNPs) (prepared with 30 mg/L AgNPs) embedded in carboxymethyl cellulose (CMC) film at 35 °C.

The principal component analysis (PCA) for L* did not reveal daily distribution of data, so brightness did not change over the 14 days of observations. Both a* and b* had a daily distribution of data from days 1 to 14 (Figure 5). Besides, the bar graphs indicate the changes in a* and b*: a* became more positive over the experiment, indicating a red color change over time; b* also became more positive, indicating a color change close to yellow (Figure 5b). Therefore, the film indicators were close to a red color the longer they were exposed at 35 °C; these data are consistent with the images in Figure 4.

### 3.2. Morphology of PDA/AgNP-Embedded CMC Films

**Figure 6:**
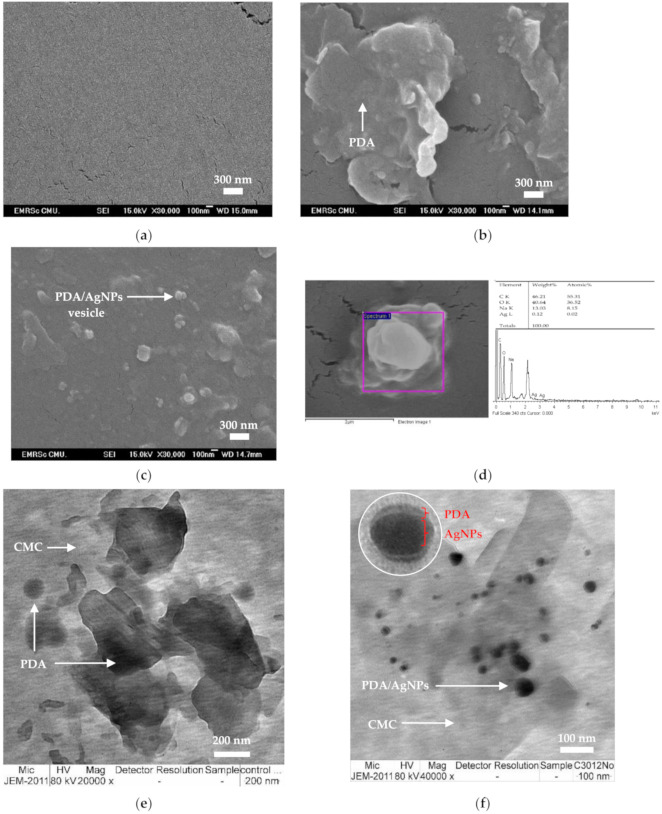
Field emission scanning electron microscopy (FE-SEM) and transmission electron microscopy (TEM) micrographs of cross-sections of (**a**) carboxymethyl cellulose (CMC) film, (**b**) polydiacetylene (PDA), and (**c**) PDA/silver nanoparticles (AgNPs) embedded in CMC film. (**d**) Energy-dispersive X-ray spectroscopy (EDS) of PDA/AgNPs. Micrographs of (**e**) PDA and (**f**) PDA/AgNPs in CMC solution.

FE-SEM was used to examine the cross-section surface area of CMC film, PDA- and PDA/AgNP- embedded CMC films. The surface of the CMC was homogeneous and smooth as shown in Figure 6a. The micrographs showed PDA phases and PDA vesicles distributed in CMC film (Figure 6b). By contrast, the PDA/AgNPs were smaller than the PDA vesicles and there were more distributed in the CMC film (Figure 6c). This finding is consistent with the size of AgNPs (20–100 nm). Energy-dispersive X-ray spectroscopy (EDS) of PDA/AgNP vesicles showed the following atomic percent values: C, 55.31%; O, 36.52%; and Ag, 0.02% (Figure 6d). With TEM, the PDA in CMC solution alone formed PDA phases and PDA vesicles with a diameter between 100 and 300 nm (Figure 6e). By contrast, the PDA/AgNP vesicles were 20–50 nm in the CMC solution (Figure 6f). The PDA vesicles coated the surface of AgNPs to form PDA/AgNP core-shell structures (Figure 6f). Marina Alloisio and co-researchers found that the final supramolecular architecture of colloids is the arrangement of the PCDA within the single bilayer [19]. An AgNP forms a sphere-like structure surrounded by PDA because functional groups incorporated between AgNPs have high affinities for the carboxylic groups in PDA [32].

**Figure 7:**
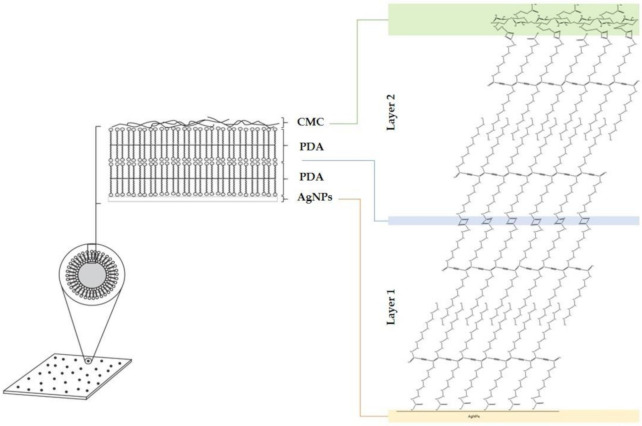
Illustration of interactions between –COOH groups of polydiacetylene (PDA) and silver (Ag)–OH interaction with carboxymethyl cellulose (CMC) at the surface of silver nanoparticles.

Besides, AgNPs are positively charged, and there are hydrogen bonds that could interact with the carboxylate of the PDA head group by ionic interactions (Figure 7). The PDA structure typically has intermolecular-type hydrogen bonds that allow it to be absorbed on the surface of AgNPs [28]. The AgNPs interacted with PDA and separated it from the phase of PDA-only structures. Thus, the mobility of PDA increased due to the augmented PDA/AgNP surface area.

### 3.3. Characterization of PDA/AgNP-Embedded CMC Films

**Figure 8:**
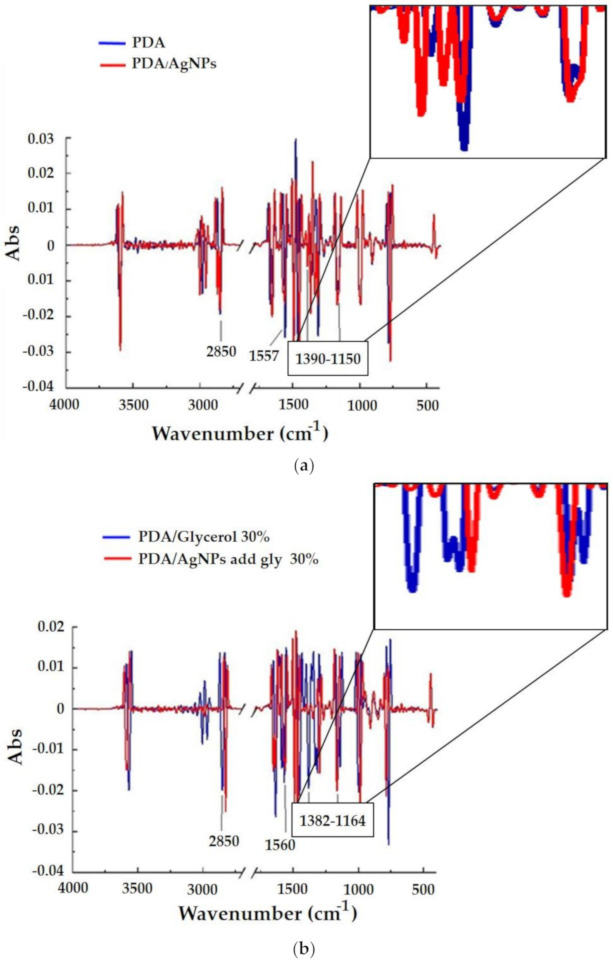
Second derivative Fourier-transform infrared (FT-IR) spectra of polydiacetylene (PDA), and the 1:3 PDA/silver nanoparticle (AgNP) ratio (prepared with 30 mg/L AgNPs) (**a**) without and (**b**) with 30% glycerol embedded in carboxymethyl cellulose (CMC) film.

FT-IR spectroscopy identified the functional groups of PDA/AgNPs in CMC films (Table 2). The signatures associated with hydrocarbon side chains are asymmetric methylene stretching (νs CH_2_) at 2850 cm^−1^, which indicates the alkyl chain conformation of blue-colored PDA [33] and is associated with the assembly of carboxyl-terminated vesicular PDA [34]. Bands at 1560–1540 cm^−1^ are usually attributed to carboxylic derivatives adsorbed on an Ag surface [28]. Symmetrical carbonyl stretching (νs C=O) is assembled with the bands corresponding to methylene bending modes [δ(CH_2_)] at 1400– 1150 cm^−1^. This band is associated with packed, bi-layered structures of carboxyl-endowed PDA [34]. Figure 8 shows the second derivative FT-IR spectra, which we used to clearly identify and easily compare the FTIR spectra of the PDA and PDA/AgNPs embedded in CMC films with and without glycerol. The differences between PDA/AgNPs and PDA/AgNPs with 30% glycerol were 1390–1150 cm^−1^ and 1382–1164 cm^−1^ [δ(CH_2_)], respectively (Figure 8a,b). The peak at 1390–1150 cm^−1^ for PDA alone (blue line) was less than for PDA/AgNPs (red line) (Figure 8a), so the PDA alone did not show a bi-layer structure. Besides, the PDA/AgNP without glycerol (Figure 8a) had peaks (red line) that were greater than PDA/AgNPs with 30% glycerol (Figure 8b), because the high glycerol concentration inhibits formation of the PDA bi-layer.

### 3.4. A Dynamic Parameter of a TTI Prototype Based on PDA/AgNP-Embedded CMC Film

**Figure 9:**
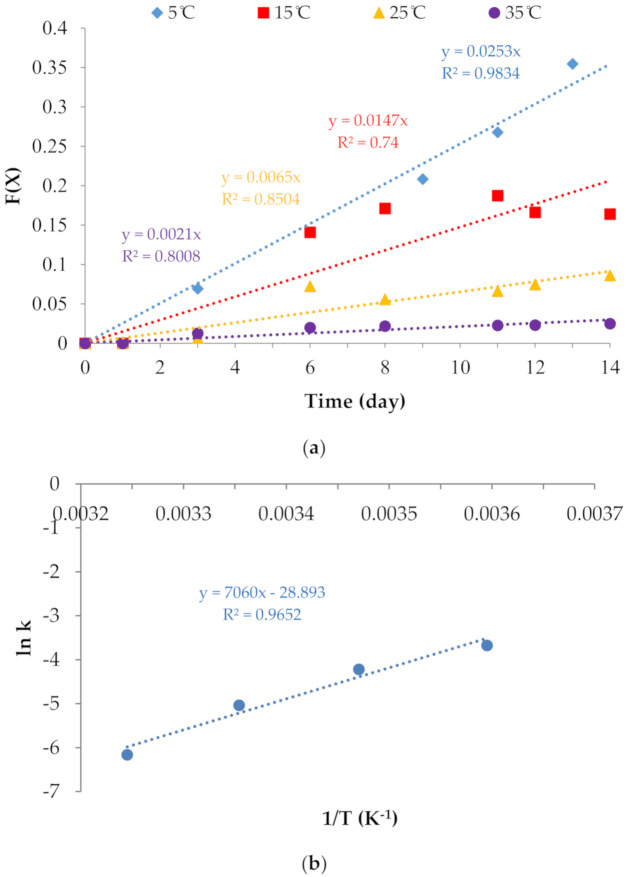
(**a**) A plot of the response function F(X) with time showed second-order kinetics at four different temperatures. (**b**) An Arrhenius plot of the response function F(X) for the total color difference showed second-order kinetics at four different temperatures.

According to Equation (2), regression lines with greater slopes were functions of the F(X) value and time (day) at different temperatures to which PDA/AgNP-embedded CMC films were exposed (Figure 9a). The indicator kinetics of the PDA/AgNP-embedded CMC film are second order. Following Equation (3), the activation energy (E_a_) of PDA/AgNP-embedded CMC film can be calculated by plotting a curve of the Arrhenius function between ln k and 1/T (Figure 9b). The E_a_ value of the TTI was 58.70 kJ/mol. The TTI based on PDA/AgNP-embedded CMC film could be used to monitor food quality losses. Poças et al. [35] reported the typical Ea values of respiration rates that occur in fruits and vegetables were in the range of 29–79 kJ/mol. Furthermore, the TTI based on PDA/AgNP-embedded CMC film could be applied to show the time–temperature of some foods: The values for fresh apples, whole carrots, lettuce, strawberries, and mushrooms were 29, 55, 37, 39, and 63 kJ/mol, respectively. If there are >± 25 kJ/mol Ea differences between food and TTIs, the accuracy of the indicator is regarded as poor [36].

## 4. Conclusions

AgNPs increased the sensitivity of the temperature-induced PDA color change. The PDA/AgNP- embedded CMC film exhibited a color change below 60 °C due to the interaction between PDA and AgNPs. Indeed, the AgNPs are good thermal conductors and help expose PDA to temperature changes. Besides, AgNPs can increase the surface area of PDA by breaking up the bulky structure of PDA alone and increasing PDA mobility. However, adding glycerol inhibited formation of a PDA/AgNP bi-layer structure. Based on the E_a_, PDA/AgNP-embedded CMC film can serve as a TTI for fruits and vegetables. For the future, PDA/AgNP-embedded CMC films have excellent potential to be developed as active and intelligent packaging systems that can inhibit microorganisms and be effective TTIs for food quality monitoring applications.

## Figures and Tables

**Table 1:** The ratios of reagents [carboxymethyl cellulose (CMC)/ Polydiacetylene (PDA)/Silver Nanoparticles (AgNPs)] examined in this study.

CMC:PDA/AgNPs (Volume:Volume)	PDA (Volume)	AgNPs (Volume)	Concentration of AgNPs (mg/L)
**1:1**	1	3	0, 30, 60, 90 and 120
	2	2	0, 30, 60, 90 and 120
	3	1	0, 30, 60, 90 and 120
**2:1**	1	3	0, 30, 60, 90 and 120
	2	2	0, 30, 60, 90 and 120
	3	1	0, 30, 60, 90 and 120
**3:1**	1	3	0, 30, 60, 90 and 120
	2	2	0, 30, 60, 90 and 120
	3	1	0, 30, 60, 90 and 120

**Table 2:** Vibration modes and band frequencies in polydiacetylene (PDA) and PDA/silver nanoparticles (AgNPs) embedded in CMC film.

Sample	Chemical Group	Wavenumber (cm^−1^)	References
PDA	CH_2_ from the alkyl chain conformation and the carboxyl-terminated	2850	[29]
PDA/AgNPs	C=O from carboxylic derivatives	1560–1540	[31]
PDA, PDA/AgNPs	CH_2_ from carbonyl group	1400–1150	[26,31]

## References

[1] Shimoni E., Anderson E., Labuza T.P. (2001). Reliability of time-temperature indicators under temperature abuse. J. Food Sci..

[2] Wandel M., Bugge A. (1997). Environmental concern in consumer evaluation of food quality. Food Qual. Pref..

[3] Giannakourou M., Koutsoumanis K., Nychas G., Taoukis P. (2005). Field evaluation of the application of time temperature integrators for monitoring fish quality in the chill chain. Int. J. Food Microbiol..

[4] Kim M.J., Jung S.W., Park H.R., Lee S.J. (2012). Selection of an optimum pH-indicator for developing lactic acid bacteria-based time–temperature integrators (TTI). J. Food Eng..

[5] Nopwinyuwong A., Kaisone T., Hanthanon P., Nandhivajrin C., Boonsupthip W., Pechyen C., Suppakul P. (2014). Effects of nanoparticle concentration and plasticizer type on colorimetric behavior of polydiacetylene/silica nanocomposite as time-temperature indicator. Energy Procedia.

[6] Jelinek R., Ritenberg M. (2013). Polydiacetylenes–recent molecular advances and applications. RSC Adv..

[7] Nopwinyuwong A., Boonsupthip W., Pechyen C., Suppakul P. (2013). Formation of polydiacetylene/silica nanocomposite as a colorimetric indicator: Effect of time and temperature. Adv. Polymer Technol..

[8] Traiphol N., Rungruangviriya N., Potai R., Traiphol R. (2011). Stable polydiacetylene/ZnO nanocomposites with two-steps reversible and irreversible thermochromism: The influence of strong surface anchoring. J. Colloid Interface Sci..

[9] Kwon Y.K., Jung J.M., Lee K.H. (2007). Preparation of polydiacetylene-attached TiO_2_ nanoparticles. Mol. Cryst. Liq. Cryst..

[10] Alloisio M., Zappia S., Demartini A., Ottonelli M., Dellepiane G., Thea S., Zoppi A., Giorgetti E., Muniz-Miranda M. (2012). Novel polydiacetylene-functionalized nanostructures for sensing applications. e-J. Surf. Sci. Nanotechnol..

[11] Won S.H., Sim S.J. (2012). Signal enhancement of a micro-arrayed polydiacetylene (PDA) biosensor using gold nanoparticles. Analyst.

[12] Nopwinyuwong A., Kitaoka T., Boonsupthip W., Pechyen C., Suppakul P. (2014). Effect of cationic surfactants on characteristics and colorimetric behavior of polydiacetylene/silica nanocomposite as time–Temperature indicator. Appl. Surf. Sci..

[13] Wang Y.-C., Lu L., Gunasekaran S. (2017). Biopolymer/gold nanoparticles composite plasmonic thermal history indicator to monitor quality and safety of perishable bioproducts. Biosens. Bioelectron..

[14] Kanmani P., Rhim J.-W. (2014). Physicochemical properties of gelatin/silver nanoparticle antimicrobial composite films. Food Chem..

[15] Shen W., Zhang X., Huang Q., Xu Q., Song W. (2014). Preparation of solid silver nanoparticles for inkjet printed flexible electronics with high conductivity. Nanoscale.

[16] Sintubin L., Verstraete W., Boon N. (2012). Biologically produced nanosilver: Current state and future perspectives. Biotechnol. Bioeng..

[17] Prema P., Thangapandiyan S., Immanuel G. (2017). CMC stabilized nano silver synthesis, characterization and its antibacterial and synergistic effect with broad spectrum antibiotics. Carbohydr. Polym..

[18] Schmidt G., Malwitz M.M. (2003). Properties of polymer–nanoparticle composites. Curr. Opin. Colloid Interface Sci..

[19] Alloisio M., Zappia S., Demartini A., Espinoza M.I.M., Ottonelli M., Dellepiane G., Thea S., Cavalleri O., Rolandi R. (2015). Silver-polydiacetylene core–shell nanohybrids: From nano to mesoscale architectures. Nano Struct. Nano Objects.

[20] Zheng M., Gu M., Jin Y., Jin G. (2001). Optical properties of silver-dispersed PVP thin film. Mater. Res. Bull..

[21] Avella M., de Vlieger J.J., Errico M.E., Fischer S., Vacca P., Volpe M.G. (2005). Biodegradable starch/clay nanocomposite films for food packaging applications. Food Chem..

[22] Tang X., Alavi S. (2011). Recent advances in starch, polyvinyl alcohol based polymer blends, nanocomposites and their biodegradability. Carbohydr. Polym..

[23] Yadav M., Rhee K., Jung I., Park S. (2013). Eco-friendly synthesis, characterization and properties of a sodium carboxymethyl cellulose/graphene oxide nanocomposite film. Cellulose.

[24] Su J.-F., Huang Z., Yuan X.-Y., Wang X.-Y., Li M. (2010). Structure and properties of carboxymethyl cellulose/soy protein isolate blend edible films crosslinked by Maillard reactions. Carbohydr. Polym..

[25] Ahmad M.B., Lim J.J., Shameli K., Ibrahim N.A., Tay M.Y., Chieng B.W. (2012). Antibacterial activity of silver bionanocomposites synthesized by chemical reduction route. Chem. Cent. J..

[26] Qian X., Staädler B. (2019). Recent developments in polydiacetylene-based sensors. Chem. Mater..

[27] Gu Y., Cao W., Zhu L., Chen D., Jiang M. (2008). Polymer mortar assisted self-assembly of nanocrystalline polydiacetylene bricks showing reversible thermochromism. Macromolecules.

[28] Chen X., Li J., Pang S., Shen A., Jiang L. (1999). Generation of two-dimensional ordered domains of gold mediated by pentacosadiynoic acid. Surf. Sci..

[29] Carpick R.W., Sasaki D.Y., Marcus M.S., Eriksson M., Burns A.R. (2004). Polydiacetylene films: A review of recent investigations into chromogenic transitions and nanomechanical properties. J. Phys. Condens. Matter.

[30] Tong Q., Xiao Q., Lim L.T. (2013). Effects of glycerol, sorbitol, xylitol and fructose plasticisers on mechanical and moisture barrier properties of pullulan–alginate–carboxymethylcellulose blend films. Int. J. Food Sci. Technol..

[31] Gou M., Guo G., Zhang J., Men K., Song J., Luo F., Zhao X., Qian Z., Wei Y. (2010). Time–temperature chromatic sensor based on polydiacetylene (PDA) vesicle and amphiphilic copolymer. Sens. Actuators B Chem..

[32] Alloisio M., Demartini A., Cuniberti C., Dellepiane G., Muniz-Miranda M., Giorgetti E. (2008). Spectroscopical investigation on colloidal suspensions of diacetylene-capped gold nanoparticles. Vib. Spectrosc..

[33] Kew S.J., Hall E.A. (2006). pH response of carboxy-terminated colorimetric polydiacetylene vesicles. Anal. Chem..

[34] Poças M.F., Delgado T., Oliveira F.A., Kerry J., Butler B. (2008). Smart packaging technologies for fruits and vegetables. Smart Packaging Technologies for Fast Moving Consumer Goods.

[35] Taoukis P., Koutsoumanis K., Nychas G. (1999). Use of time–temperature integrators and predictive modelling for shelf life control of chilled fish under dynamic storage conditions. Int. J. Food Microbiol..

[36] Seo D., Kim J. (2010). Effect of the molecular size of analytes on polydiacetylene chromism. Adv. Funct. Mater..

